# Stroke epidemiology and outcomes of stroke patients in Nepal: a systematic review and meta-analysis

**DOI:** 10.1186/s12883-023-03382-5

**Published:** 2023-09-25

**Authors:** Raju Paudel, Christine Tunkl, Shakti Shrestha, Ram Chandra Subedi, Ayush Adhikari, Lekhjung Thapa, Bikram Prasad Gajurel, Avinash Chandra, Ghanashyam Kharel, Pankaj Jalan, Subash Phuyal, Babu Ram Pokharel, Subi Acharya, Kanchan Bogati, Pinky Jha, Naresh Kharbuja, Christoph Gumbinger

**Affiliations:** 1 Grande International Hospital, Kathmandu, Nepal; 2 University Hospital Heidelberg, Heidelberg, Germany; 3 University of Queensland, Queensland, Australia; 4 Tribhuvan Univerisity Teaching Hospital, Kathmandu, Nepal; 5 National Neuro Center, Kathmandu, Nepal; 6 Annapurna Neurological Institute and Allied Sciences, Kathmandu, Nepal; 7 National Institute of Neurological and Allied Sciences, Kathmandu, Nepal; 8 Norvic International Hospital, Lalitpur, Nepal; 9 Nepal Mediciti, Lalitpur, Nepal; 10 Patan Academy of Health Sciences, Lalitpur, Nepal; 11 Nepalese Army Institute of Health Sciences, Kathmandu, Nepal

**Keywords:** Epidemiology, Outcomes, Review, Stroke

## Abstract

**Background:**

With an increasing burden of stroke, it is essential to minimize the incidence of stroke and improve stroke care by emphasizing areas that bring out the maximum impact. The care situation remains unclear in the absence of a national stroke care registry and a lack of structured hospital-based data monitoring. We conducted this systematic review and meta-analysis to assess the status of stroke care in Nepal and identify areas that need dedicated improvement in stroke care.

**Methods:**

A systematic literature review was conducted to identify all studies on stroke epidemiology or stroke care published between 2000 and 2020 in Nepal. Data analysis was done with Statistical Package for Social Sciences (SPSS) and Comprehensive Meta-analysis (CMA-3).

**Results:**

We identified 2533 studies after database searching, and 55 were included in quantitative and narrative synthesis. All analyses were done in tertiary care settings in densely populated central parts of Nepal. Ischemic stroke was more frequent (70.87%) than hemorrhagic (26.79%), and the mean age of stroke patients was 62,9 years. Mortality occurred in 16.9% (13-21.7%), thrombolysis was performed in 2.39% of patients, and no studies described thrombectomy or stroke unit care.

**Conclusion:**

The provision of stroke care in Nepal needs to catch up to international standards, and our systematic review demonstrated the need to improve access to quality stroke care. Dedicated studies on establishing stroke care units, prevention, rehabilitation, and studies on lower levels of care or remote regions are required.

**Supplementary Information:**

The online version contains supplementary material available at 10.1186/s12883-023-03382-5.

## Background

Stroke has become a critical global public health challenge requiring prompt and effective intervention. In particular,12.2 million new cases, 101 million prevalent cases, and 6.55 million stroke-related deaths were reported [1]. The data show a remarkable increase in stroke incidence and mortality rates from 1990 to 2019, with a 70% rise in stroke incidence and a 43% rise in stroke-related deaths [1].

The situation of stroke in Asian countries is not different from the global scenario. The reported incidence of stroke in Asia ranges from 116 to 483 per 100,000 per year [2–4]. Furthermore, evidence suggests that South Asians have a twofold higher risk of getting a stroke than Europeans due to the higher prevalence of dyslipidemia, diabetes mellitus, and central obesity [5, 6]. Nepal, a South Asian country with a population of 29 million, has reported a relatively high crude and age-standardized prevalence of stroke in the southwestern region in 2018, with rates of 2368 and 2967 per 100,000 population, respectively [7]. However, this data only represents a specific region and may not be generalizable to the Nepalese context [2].

Improving stroke care demands reliable data on stroke epidemiology, risk factors, treatment, and outcomes. However, such data are not available for Nepal. Therefore, this systematic review aims to fill this knowledge gap by exploring stroke studies conducted in the Nepalese population regarding stroke epidemiology, risk factors, treatment, and outcomes. This review will help to identify the needs in stroke care in Nepal.

## Methods

This systematic review was conducted following the Preferred Reporting Items for Systematic Reviews and Meta-Analysis (PRISMA) statement. The study protocol was registered in PROSPERO [8] prior to the conduct of the review.

### Selection of studies

#### Inclusion criteria

We included studies published in English between January 1, 2000, and January 1, 2021, reporting empirical data (quantitative, qualitative) obtained in Nepal. Studies were included if participants had a confirmed stroke diagnosis and were at least 18 years of age. (REF: Sacco L et al., Stroke, 2013) or TIA (REF: Easton et al., Stroke, 2009) and reported on incidence, epidemiology, risk factors, etiology, stroke outcome, or stroke treatment (e.g., diagnosis, acute and post-acute care, rehabilitation, financing of stroke care, complications of stroke).

#### Exclusion criteria

We excluded articles that could not be classified as empirical literature (e.g., commentaries, discussion papers, journalistic interviews, policy reports), reviews, studies on stroke mimics (e.g., migraine), and studies on mixed populations (e.g., South Asians) unless separate results for people with stroke in Nepal could be isolated. Studies reporting on adults < 18 years were excluded.

### Search strategy

The study followed the “Cochrane Guidelines for Systematic Reviews of Health Promotion and Public Health Interventions” in designing the search strategy. PubMed, Ovid, Cochrane Library, Web of Science, and clinicaltrials.org were searched for English-language articles published between 2000 and 2020. Google and Google Scholar identified grey literature not indexed in academic databases was identified. The search terms and keywords related to stroke, knowledge, epidemiology, and treatment. The ‘Appendix 1 Search strategy’ contains the detailed search strategy. Additionally, the reference lists of included papers were screened.

### Study selection

Study selection was performed by (1) independent screening of titles and abstracts (RP, CT), and (2) Independent screening of full texts of all hits judged suitable in the first step (RP, CT). Discrepant ratings were discussed and agreed upon in consensus meetings (AC, BPG, LT, and PJ). Specificity (proportion of suitable articles in all hits) and sensitivity (proportion of suitable articles in all correct positives) were calculated as quality criteria for the search strategy based on a predefined test set. Subsequently, studies on stroke awareness were excluded to keep the review topic specific to stroke care.

### Data extraction, synthesis, and analysis

A data extraction form was designed, including author, year, study title, sample characteristics, stroke prevalence, incidence, etiology, risk factors, treatment (recanalization therapy, length of hospital stay), mortality, complications, outcome, and diagnostic findings. The included articles were extracted by AA, KB, SA, and PJ and checked by RP and CT. Given the significant heterogeneity of the included studies, a narrative data synthesis was performed. The heterogeneity of the studies was calculated using the I2 statistics; for I2 ≤ 50, a fixed effects model was used. For I2 > 50, the random effects model was used and represented using forest plots with CMA-3 for meta-analysis and SPSS 22 for descriptive analysis. The quality of the included studies was assessed using the Oxford Centre of Evidence-Based Medicine: Level of Evidence (March 2009) [9].

## Results

### Study selection

**Fig. 1:**
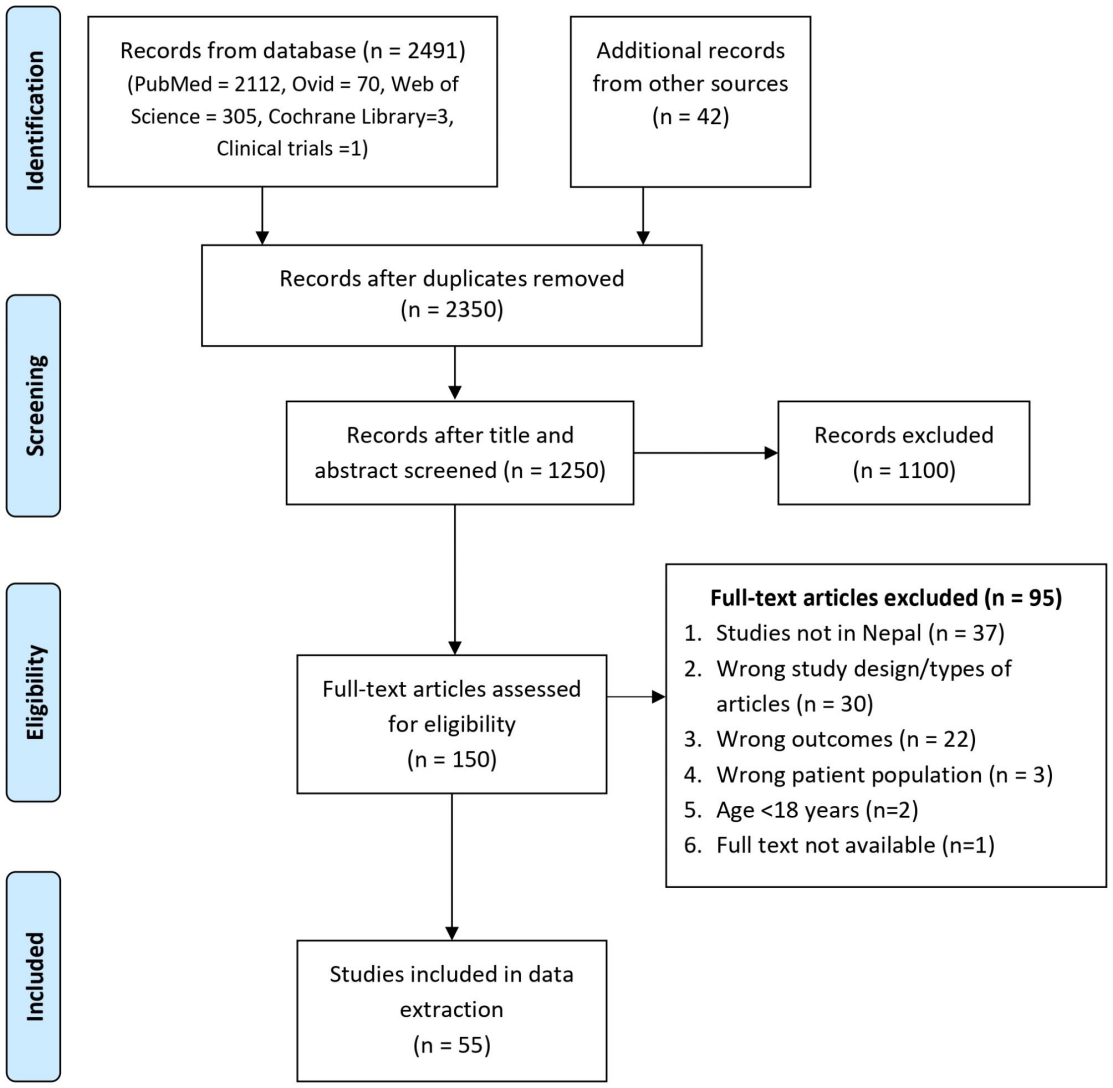
PRISMA Flow Diagram

A total of 2533 studies were identified, and 141 duplicates were removed. The title and abstracts of 1250 studies were screened, and 1100 studies were excluded. Full texts of 150 studies were assessed, and 95 studies were excluded for definite reasons. A total of 55 studies were included in this systematic review (Fig. 1). Specificity was 3.9%, and sensitivity was 100%.

#### Study characteristics

Among the 55 included quantitative studies on stroke patients in Nepal, 19 were cross-sectional studies [10–28], 17 were retrospective [29–45], and 17 were prospective cohort studies [46–62]. Two case-control studies were present [63, 64], while no randomized trials were found. The sample size of quantitative studies ranged from nine to 1017 participants. Studies were mainly published in national journals (n = 48) [10–21, 23–35, 37, 39, 41–43, 45–56, 58–60, 63, 64], and only seven studies were published in international journals [22, 36, 40, 44, 57, 61, 62].

#### Study population

Thirty-four studies reported the mean age of the patient population [10, 12–14, 19–23, 26–28, 30, 31, 33, 36–41, 45, 48, 50–53, 56, 57, 59, 60, 62–64]. As indicated in Table 1, the pooled mean age was 62.4 years, ranging from 51.9 [38] to 70.5 years [18]. From 46 studies reporting data on sex distribution, 44 studies showed a higher percentage of stroke in males [10, 12–14, 16, 19–21, 23–28, 30–33, 35–41, 43–45, 47–51, 53–59, 61–64] (Table 1).

**Table 1:** Demographics and types of strokes

Title	Total
Total number of patients	7499
Mean age in years	62.44
Sex (Male/Female, % male)	3441/2303, 59.90%
Types (Ischemic/Hemorrhagic)	5294/2001

#### Study settings and location

**Fig. 2:**
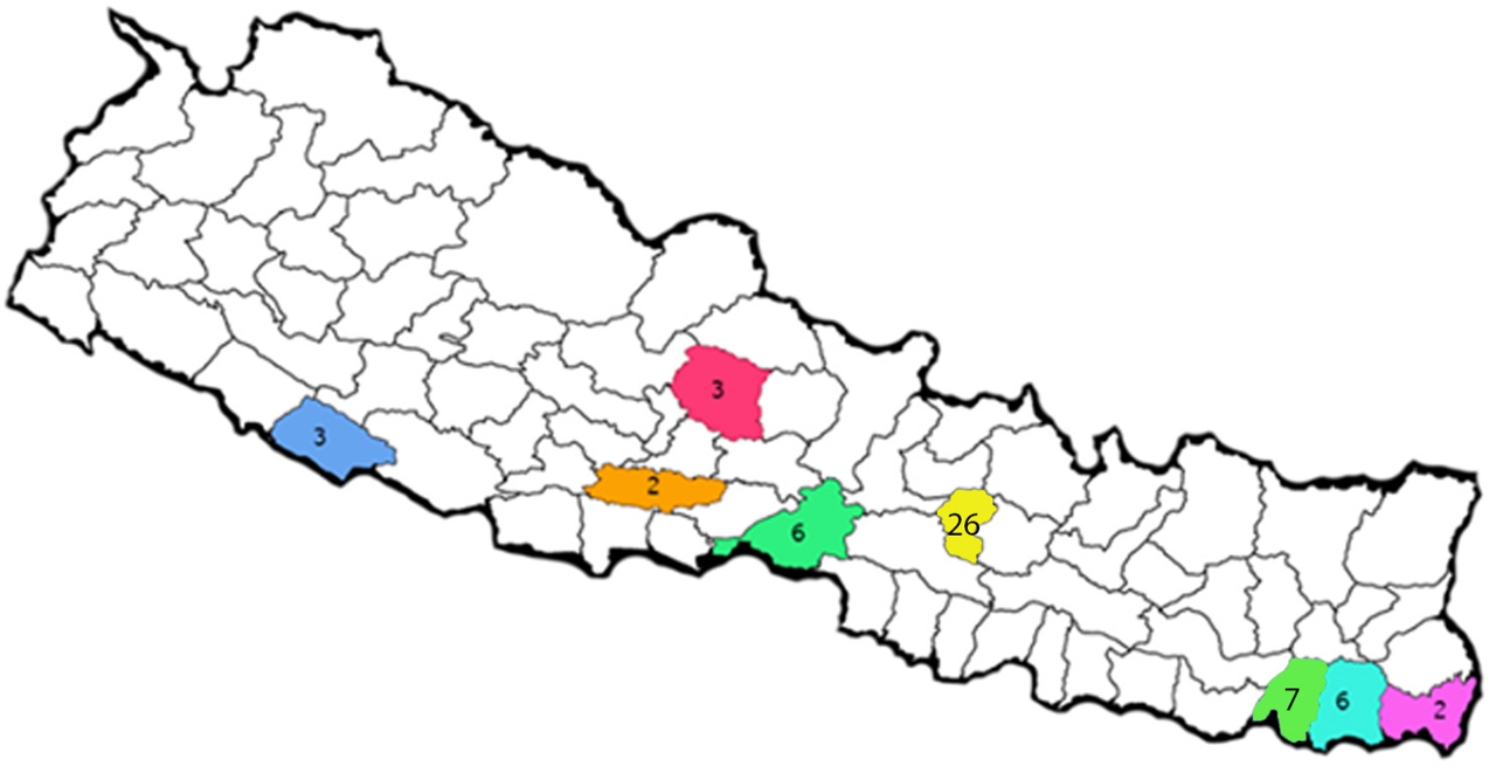
District map of Nepal and the number of studies done in those districts

All 55 studies were hospital-based and were conducted in the tertiary health sector. Of the 55 studies in stroke patients, majority (n = 26) were done in Kathmandu valley [12, 13, 15, 16, 19, 22, 27–29, 31, 33, 34, 41–45, 49–52, 54, 55, 59, 61, 64], followed by Sunsari (n = 7) [18, 30, 40, 53, 56, 60, 63], Chitwan (n = 6) [21, 25, 26, 47, 48, 57] and Morang (n = 6) [24, 32, 36, 46, 58, 62], which represent Central and Eastern region of Nepal. Only three studies were done in Nepalgunj, the Mid-western part of Nepal [11, 23, 39]. The location of each study within Nepal is presented in Table 2 and represented in Fig. 2.

**Table 2:** Summary of included studies

Lead author (year)	Setting	Sample size	Age in years; % female	Comorbidities	Main focus areas
Acharya S. et al. 2016 [10]	Secondary care, Lumbini	200	62.2 ± 12.7; 38.0%	59.0% HTN; 39.0% DM; 61.0% Smoking; 8.0% Previous stroke	Clinico-radiological profile
Acharya SP. et al. 2018 [29]	Tertiary care, KTM	11	*Data NA*	*Data NA*	Profile of neurological patients
Acharya S. et al. 2014 [11]	Secondary care, Lumbini	71	*Data NA*	*Data NA*	Use of CT scan in stroke
Adhikari J. et al. 2019 [30]	Tertiary care, Dharan	278	65.0 ± 15.0; 38.8%	57.2% HTN; 25.2% DM; 38.5% Smoking; 4.0% Alcohol; 14.4% Previous stroke	Complications and mortality
Aryal M. et al. 2010 [31]	Tertiary care, KTM	82	62.4; 30.5%	50.0% HTN; 9.8% DM	Management
Bhatt VR et al. 2008 [12]	Tertiary care, KTM	61	61.0; 47.5%	73.8% HTN; 34.4% DM; 57.4% Smoking; 47.5% Alcohol; 21.3% HLD; 4.9% AF; 6.6% Previous stroke;	Risk factors
Cherian I. et al. 2018 [32]	Secondary care, Biratnagar	102	*Age Data NA*; 47.1%	42.2% Smoking; 15.7% Alcohol	ICH and surgical options
Chhetri PK. et al. 2012 [47]	Secondary care, Bharatpur	100	*Age Data NA*; 28.0%	28.0% HTN; 3.0% DM; 38.0% Smoking; 13.0% Alcohol	CT scan in stroke
Deo RK. et al. 2008 [63]	Tertiary care, Dharan	25	61.8 ± 11.1; 40%	84.0% HTN; 100% DM; 52.0% Smoking	DM and stroke
Devkota KC. et al. 2006 [33]	Secondary care, KTM	72	61.7 ± 14.9; 41.7%	47.2% HTN; 11.1% DM; 58.3% Smoking; 40.3% Alcohol; 12.5% AF	Risk factors
Dewan KR. et al. 2014 [48]	Secondary care, Bharatpur	100	67.15 ± 12.6; 34%	72.0% HTN; 19.0% DM; 66.0% Smoking; 43.0% Alcohol; 53.0% HLD; 25.0% AF	Mortality and risk factors
Dhungana K et al. 2018 [49]	Tertiary care, KTM	180	*Age Data NA*; 48.9%	50% HTN; 6.1% DM; 36.1% Smoking; 8.3% Previous stroke	Demographic characteristics
Dhungana K et al. 2019 [13]	Tertiary care, KTM	96	64.4 ± 14.0; 47.9%	51% HTN; 17.7% DM; 50.0% Smoking; 21.9% Alcohol	Complications
Gajurel BP et al. 2012 [34]	Tertiary care, KTM	343	*Data NA*	*Data NA*	Neurological disorders in admitted patients
Gajurel BP et al. 2014 [50]	Tertiary care, KTM	200	61.5 ± 16.3; 46%	42.5% HTN; 14.5% DM; 58.5% Smoking; 12.5% AF	Demographics, risk factors, and outcomes
Gautam B et al. 2018 [14]	Secondary care, Tansen	71	66.0 ± 11.4; 40.8%	45.1% Smoking; 31.0% Alcohol	Besson score to distinguish non-hemorrhagic and hemorrhagic stroke
Ghimire RK et al. 2005 [64]	Tertiary care, KTM	97	64.0; 29.9%	60.8% HTN; 23.7% DM; 52.6% Smoking; 36.1% Alcohol	Carotid doppler in stroke
Jha R et al. 2018 [15]	Secondary care, KTM	34	*Data NA*	*Data NA*	Association of microalbuminuria with stroke
Jwarchan B et al. 2020 [35]	Secondary care, Pokhara	56	*Age Data NA*; 39.3%	53.6% HTN; 48.2% DM	Prevalence
Karn R et al. 2018 [51]	Tertiary care, KTM	182	65.0 ± 13.7; 42.3%	46.7% HTN; 16.5% DM; 18.7% AF; 15.4% Previous stroke	Outcomes based on TOAST classification
Karn R et al. 2015 [16]	Tertiary care, KTM	151	*Age Data NA*; 43.7%	11.9% DM	Fever in stroke
Keyal NK et al. 2020 [17]	Tertiary care, Dharan	76	*Data NA*	*Data NA*	Outcome of Neuro ICU
Khattar NK et al. 2019 [36]	Secondary care, Biratnagar	21	56.0; 23.8%	76.2% HTN; 57.1% DM; 76.2% Alcohol	Demographics
Koirala SR et al. 2016 [52]	Secondary care, KTM	181	64.2 ± 15.9; 53%	68.5% HTN; 11.6% DM; 74.6% Smoking; 14.4% HLD; 10.5% AF; 1.7% Previous stroke	Prognostic factors
Kumari S et al. 2018 [18]	Tertiary care, Dharan	52	70.5 ± 6.4	55.8% HTN; 11.5% DM; 9.6% Previous stroke	CT scan in acute confusion
Lamichane BS et al. 2020 [37]	Secondary care, Pokhara	86	64.3 ± 12.7; 30.2%	72.1% HTN; 17.4% DM; 48.8% Smoking; 25.6% HLD; 12.8% AF	Clinical profile
Luitel R et al. 2020 [19]	Secondary care, KTM	310	60.7 ± 16.3; 34.2%	67.4% HTN; 29.7% DM; 44.8% Smoking; 3.2% Alcohol	Demographics
Maskey A. et al. 2011 [20]	Secondary care, Pokhara	160	66.0 ± 10.7; 35%	61.3% HTN; 9.4% DM; 59.4% Smoking; 26.9% Alcohol; 33.8% HLD; 23.1% AF	Risk factors
Naik M et al. 2006 [53]	Tertiary care, Dharan	150	58.3 ± 16.0; 30.7%	40.0% HTN; 6.7% DM; 40.7% Smoking; 30.7% Alcohol	Clinico-radiological profile
Nepal G et al. 2019 [61]	Tertiary care, KTM	228	*Age Data NA*; 46.9%	56.1% HTN; 17.5% DM; 28.9% Smoking; 27.2% Alcohol; 11.8% Previous stroke	Thrombolysis in management
Nepal PR et al. 2020 [38]	Secondary care, Jhapa	31	51.9 ± 12.3; 25.8%	*Data NA*	Outcomes of decompressive surgery
Nepal R et al. 2020 [62]	Tertiary care, Dharan	168	69.0 ± 1; 43.5%	69% HTN; 32.7% DM; 33.9% Smoking; 14.9% Alcohol; 72.0% HLD; 71.4% AF	Prevalence, causes, and mortality
Pahari SK et al. 2013 [54]	Secondary care, KTM	28	*Age Data NA*; 39.3%	53.6% HTN; 28.6% DM; 39.3% Smoking; 71.4% HLD	Lipid profile and carotid doppler in ischemic stroke
Poudel RS et al. 2015 [21]	Tertiary care, KTM	37	63.4 ± 16.3; 27%	59.5% HTN; 13.5% DM; 73% Smoking; 51.4% Alcohol; 10.8% AF; 16.2% Previous stroke	Treatment
Rajouria AD et al. 2012 [55]	Secondary care, KTM	75	*Age Data NA*; 25.3%	*Data NA*	Usefulness of stroke scoring
Roka YB. et al. 2011 [46]	Tertiary care, Dharan	36	*Data NA*	*Data NA*	Clinical profile
Shah SK et al. 2016 [39]	Secondary care, Nepalgunj	119	59.8 ± 11.2; 42.9%	50.4% HTN; 9.2% DM; 58.8% Smoking; 63.0% Alcohol	Risk factors
Shah B et al. 2017 [40]	Tertiary care, Dharan	257	66.0 ± 14.0; 42.0%	57.6% HTN; 25.3% DM; 39.7% Smoking; 3.9% Alcohol	In-hospital mortality
Shah B et al. 2020 [56]	Tertiary care, Dharan	107	63.0 ± 15.0; 44.9%	55.1% HTN; 15.9% DM; 59.8% Smoking; 52.3% Alcohol; 22.4% Previous stroke	Mortality and outcomes
Shakya D et al. 2019 [27]	Secondary care, Karnali	155	61.6 ± 14.0; 42.6%	*Data NA*	Quality of life in stroke survivors
Shrestha A et al. 2011 [41]	Secondary care, KTM	210	58.5; 41.0%	38.6% HTN; 10.0% DM; 60.5% Smoking; 41.4% Alcohol; 9.0% HLD	Risk factors
Shrestha E et al. 2020 [28]	Secondary care, KTM	155	63.0 ± 15.0; 45.2%	30.3% HTN; 25.2% DM; 20.6% Alcohol	CT findings in stroke
Shrestha GS et al. 2012 [22]	Tertiary care, KTM	40	52.1 ± 15.7; 60%	60.0% HTN; 22.5% DM; 50.0% Smoking	Visual neglect in stroke
Shrestha S et al. 2015 [57]	Tertiary care, KTM	56	67.0 ± 13.4; 37.5%	62.5% HTN; 14.3% DM; 58.9% Smoking; 35.7% Alcohol; 7.1% HLD; 21.4% AF; 14.3% Previous stroke;	Clinical profile
Shrestha A et al. 2018 [23]	Secondary care, Nepalgunj	51	67.7 ± 10.2; 49.0%	33.3% HTN	HTN in hemorrhagic stroke
Shrestha R et al. 2018 [43]	Secondary care, KTM	12	*Age Data NA*; 16.7%	41.7% HTN; 50.0% Smoking; 50.0% Alcohol	ICH clinical profile
Shrestha R et al. 2020 [42]	Secondary care, KTM	770	*Data NA*	*Data NA*	Admission in the emergency department
Thakur MK et al. 2017 [24]	Secondary care, Biratnagar	100	*Age Data NA*; 35.0%	61.0% HTN; 24.0% DM; 59.0% Smoking; 31.0% Alcohol; 27.0% AF	ECG changes in stroke
Thapa L et al. 2014 [25]	Secondary care, KTM	60	*Age Data NA*; 43.3%	80.0% HTN; 20.0% DM; 56.7% Smoking; 35.0% Alcohol; 8.3% HLD; 6.7% AF; 10.0% Previous stroke;	Association of Vitamin D with stroke risk factors
Thapa L et al. 2016 [44]	Secondary care, KTM	9	*Age Data NA*; 44.4%	44.4% HTN; 11.1% Smoking; 22.2% Alcohol; 11.1% AF; 11.1% Previous stroke;	Thrombolysis in ischemic stroke
Thapa A et al. 2018 [45]	Secondary care, KTM	1017	55.0 ± 21.9; 39.5%	44.4% HTN; 11.1% DM; 38.6% Smoking; 3.5% Alcohol; 8.9% HLD	Risk factors
Thapa GB et al. 2013 [60]	Secondary care, KTM	54	61.9	25.9% HTN; 3.7% DM; 9.3% Previous stroke	Carotid doppler in stroke
Thapa L et al. 2013 [26]	Secondary care, KTM	44	64.0 ± 15.3; 34.1%	*Data NA*	Mortality in ICU
Tuladhar AS et al. 2012 [59]	Secondary care, KTM	45	62.0 ± 11.0; 31.1%	*Data NA*	Carotid doppler in stroke
Yadav AK et al. 2020 [58]	Secondary care, Biratnagar	79	*Age Data NA*; 32.9%	62.0% HTN; 40.5% DM; 31.6% Smoking; 26.6% HLD	Carotid doppler in stroke

**Abbreviation**: AF, Atrial Fibrillation; CT, Computer Tomography; DM, Diabetes Mellitus; ECG, Electrocardiography; HLD, Hyperlipidaemia; HTN, Hypertension; ICH, Intracranial Haemorrhage; ICU, Intensive Care Unit; NA: Not available; TOAST, Trial of Org 10,172 in Acute Stroke Treatment

**Note**: Sample size indicates total sample size. The percentage of females and comorbidities are derived from the total sample size.

#### Outcome parameters

**Fig. 3:**
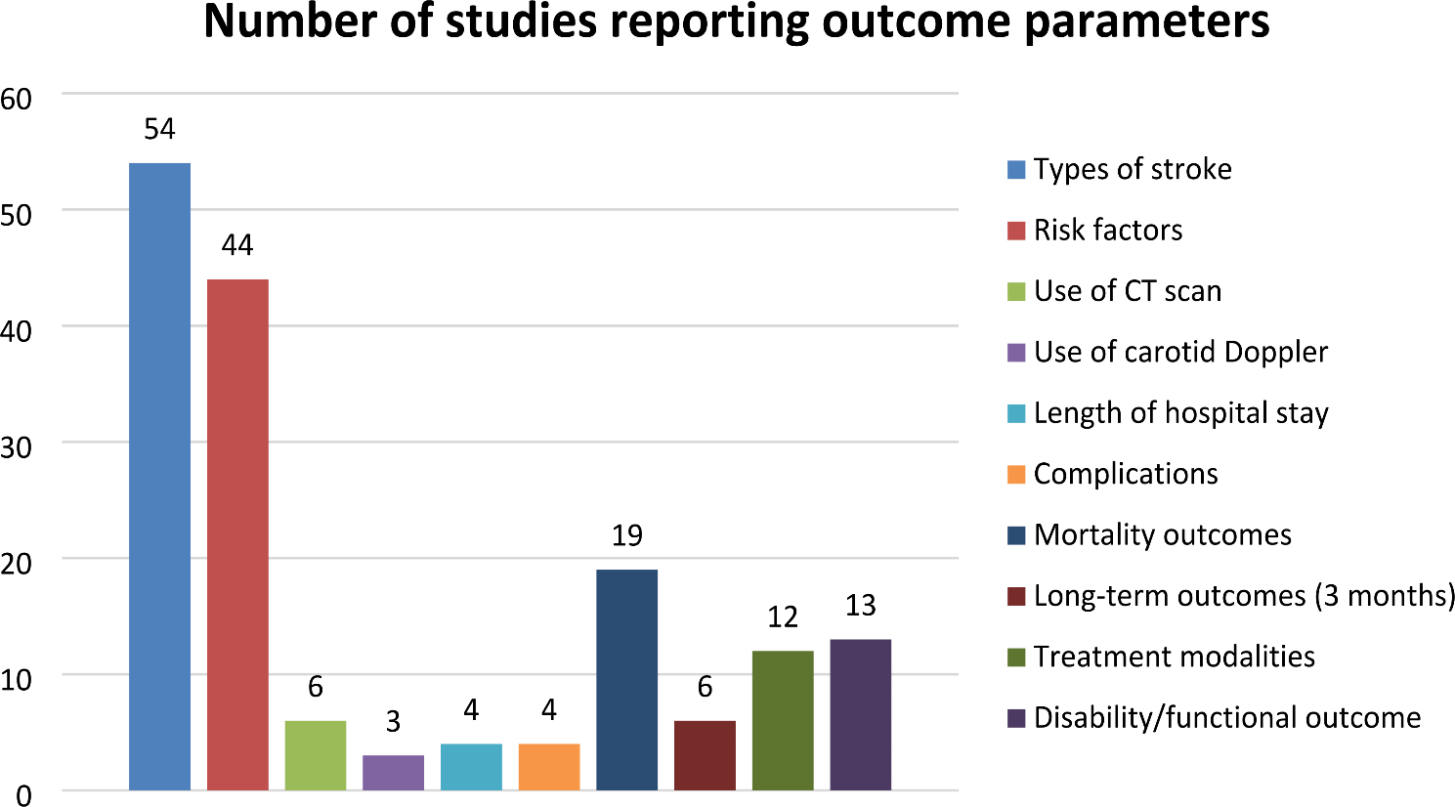
Number of studies reporting the outcome parameters

The details of the outcome parameters are presented in Table 2 under the main focus area. Fifty studies reported on the types of strokes [10–62, 64], and 44 studies investigated risk factors in stroke patients [10, 12–14, 16, 18–26, 28, 30–33, 35–37, 39–41, 43–45, 47–54, 56–59, 61–64]. Six studies described using CT scan [10, 11, 18, 28, 47, 53]. Three studies described the use of carotid Doppler in ischemic stroke [58–60]. From 23 studies investigating aspects of acute care [13, 30, 40, 49], four studies consisted of data on length of hospital stay [13, 30, 40, 49], four studies highlighted complications [13, 16, 30, 48], and 19 studies reported mortality [11, 17, 21, 26, 29, 30, 32, 34, 36, 38, 40, 41, 45, 48, 51, 52, 56, 57, 62]. 12 studies included treatment modalities [21, 31, 32, 36, 38, 42–45, 48, 57, 61] [42, 44, 61].The outcome of stroke was described in 22 studies [11, 17, 21, 26, 29, 30, 32–34, 36, 38, 40, 41, 43–45, 48, 51, 52, 56, 57, 62], and the long-term outcome (3 months after stroke) was investigated only in seven studies [21, 38, 43–45, 51, 57]. The outcome parameters of the studies have been depicted in Fig. 3.

#### Types of strokes

Fifty-Four studies reported on the types of strokes [10–62, 64]. Pooled data showed ischemic stroke in 70.87% and hemorrhagic in 26.79%. The transient ischemic attack was reported only in 0.66% of patients (Table 1).

#### Risk factors

Of 44 studies reporting risk factors in stroke patients [10, 12–14, 16, 18–26, 28, 30–33, 35–37, 39–41, 43–45, 47–54, 56–59, 61–64], pooled data showed hypertension as the most prevalent comorbidity in 50.61% of patients, followed by history of smoking (38.65%), significant alcohol intake (27.31%), diabetes (17.39%), dyslipidemia (8.59%) and atrial fibrillation (5.87%) (refer to Table 2 for more details). Other socio-economic data of patients, like ethnicity and profession, is included in ‘Appendix 3 Education, ethnicity, and job of study participants’.

#### Carotid doppler findings

Three studies reported on carotid Doppler findings in ischemic stroke patients [58–60], where 70.78% of 178 patients were found to have plaque and 18.5% had 50–99% occlusion of the carotid artery.

#### Acute and post-acute care

Data on stroke care was reported in 12 studies [13, 29, 30, 40, 48, 49], and intravenous thrombolysis (IVT) was used in 2.39% of patients [42, 44, 61], and no studies reported on endovascular thrombectomy (EVT). While no study reported on stroke unit care, the mean length of hospital stay was 6.1 days [13, 30, 40, 49]. Five studies reported the use of Aspirin with the use of Aspirin in 83.30% of patients [21, 31, 48, 57, 61]. Surgery (for hemorrhagic stroke or malignant MCA infarct) was done in 14.38% of patients.

#### Disability/functional outcomes

From 13 studies reporting on the disability and functional outcome of stroke patients, ten studies used the modified Rankin scale (mRS) [13, 21, 35, 43–45, 50, 51, 56, 57], two studies used the Glasgow outcome scale [32, 36] and one used WHO disability assessment schedule [27]. Assessment time ranged from discharge to 6 months. Most patients had mRS 3 [21, 50], and the mean average mRS ranged from 2.66 to 3.48 [50, 51]. Table 3 presents the disability and functional outcomes of 13 studies.

#### Mortality

**Fig. 4:**
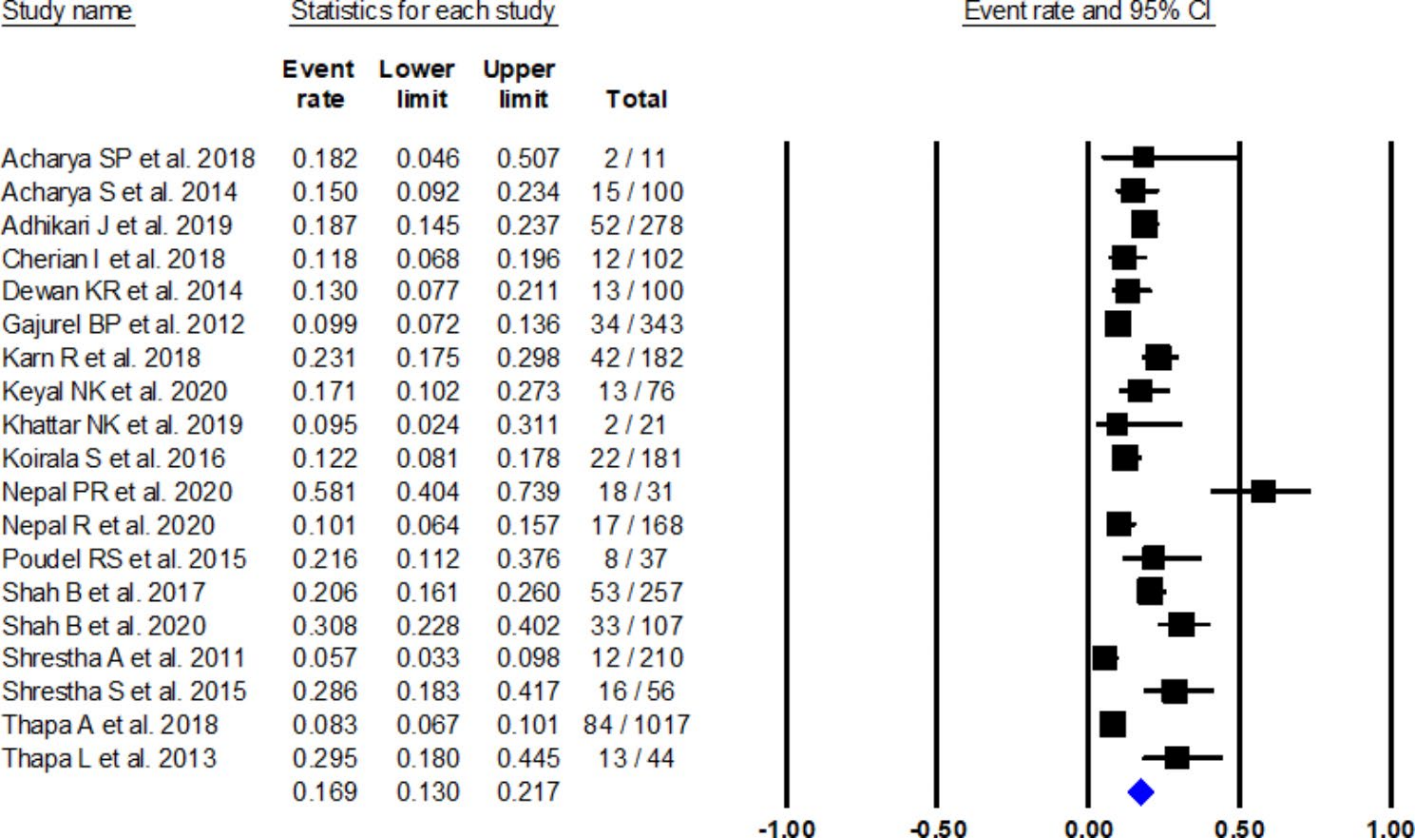
Forest plot showing mortality among stroke patients

**Fig. 5:**
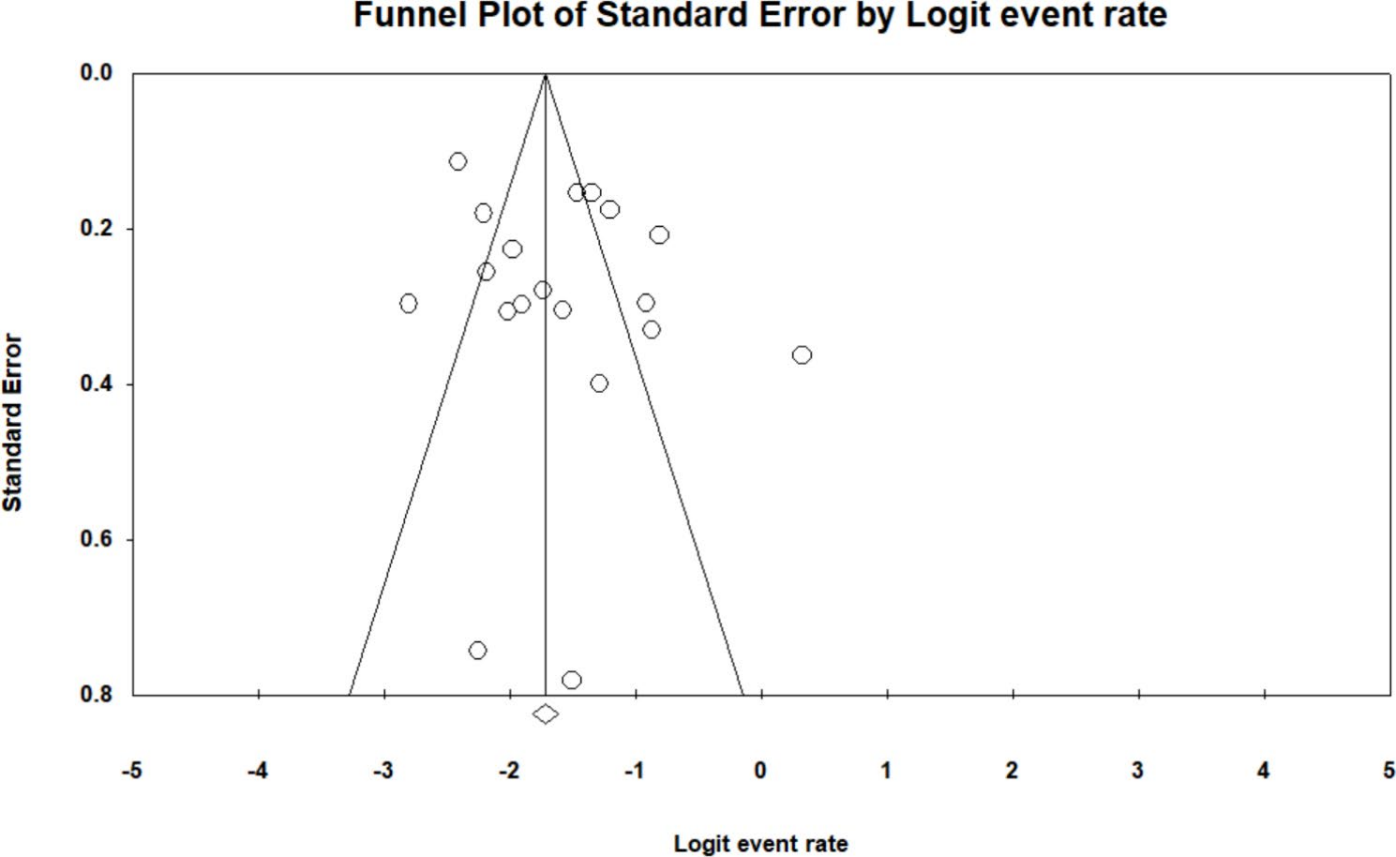
Funnel plot for detection of publication bias in meta-analysis of mortality rate in stroke patients

Mortality was reported in 19 studies [11, 17, 21, 26, 29, 30, 32, 34, 36, 38, 40, 41, 45, 48, 51, 52, 56, 57, 62]. Maximum mortality at three months was found in a study by Shrestha S et al. (28.57%), while at six months, mortality was high in a study by Nepal PR et al. (58.8%) [38, 57]. The meta-analysis showed mortality in 16.9% of patients (proportion 0.169, 95% CI: 0.130–0.217, I^2^: 88%) (see Fig. 4). Funnel plot examination and Egger’s test (P = 0.25) showed no publication bias (Fig. 5). Sensitivity analysis performed, excluding one study, did not show much change in the mortality rate. (Appendix 2 Sensitivity analysis)

#### Cost

No studies reported on the cost of stroke care or cost-effectiveness.

#### Complications

**Fig. 6:**
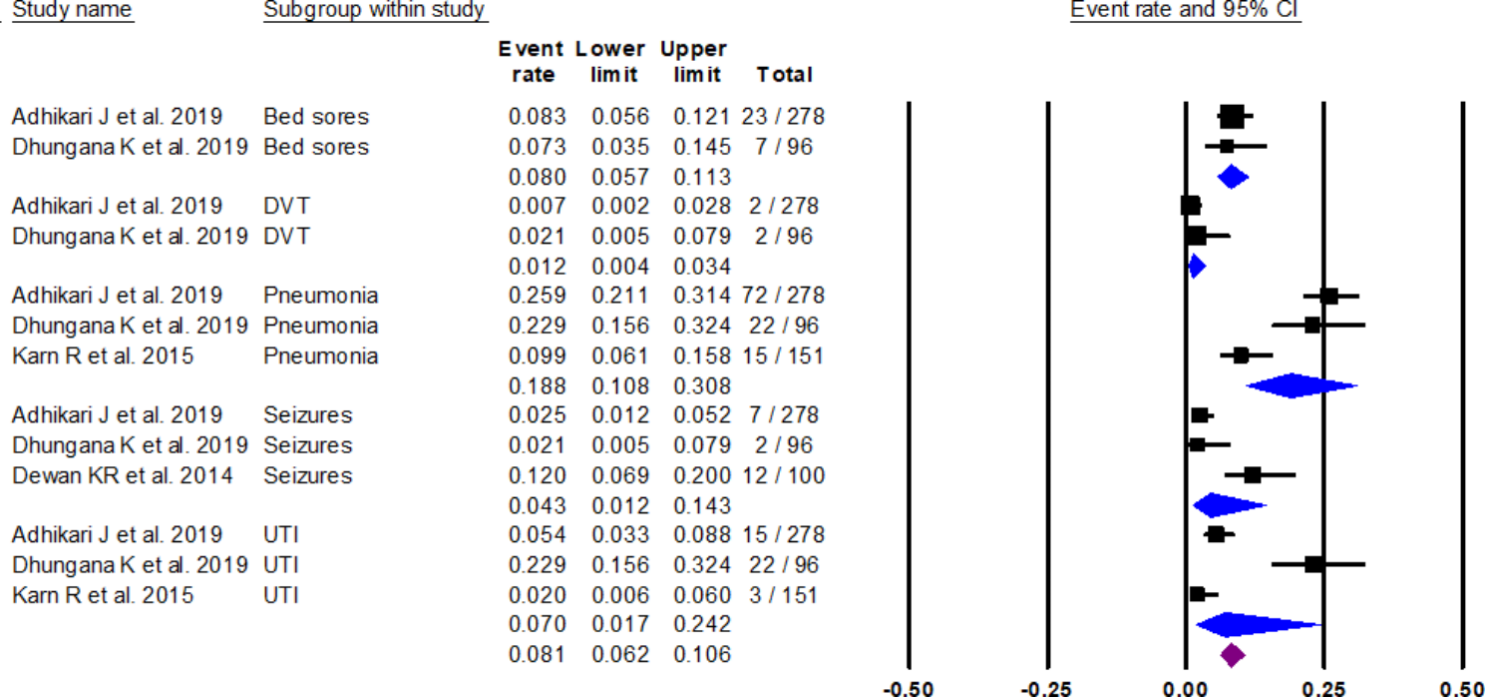
Forest plot showing complications in stroke patients

Four studies had data on the complications in stroke patients [13, 16, 30, 48] with the most common complication reported being pneumonia in 18.8% of patients (*I*2: 13.13), urinary tract infections (UTI) in 7% (*I*2: 9.24), seizures in 4.3%, and bedsores in 8% of patients. Falls, fever, and deep vein thrombosis were other reported complications (see Fig. 6).

### Quality assessment

Of the 55 studies on stroke patients, 20 were classified as having a high level of evidence (LOE 2) [19, 21–24, 27, 29, 30, 32, 35, 36, 38, 40–45, 53, 61]. Only eight studies were rated as the highest level of evidence (LOE 1) [17, 26, 46, 51, 52, 55–57]. The vast majority of studies were found to have a low level of evidence (n = 27) [10–16, 18, 20, 25, 28, 31, 33, 34, 37, 39, 47–50, 54, 58–60, 62–64].

**Table 3:** Disability and functional outcomes of the studies

STUDY	Disability/outcomes
Cherian I. et al. 2018 [32]	GOS Moderate disability at 6 weeks = 28/102 (27.5%)
Dhungana K et al. 2019 [49]	At discharge, mRS 1 = 10/96 mRS 2 = 36/96 mRS 3 = 35/96 mRS 4 = 11/96 mRS 5 = 4/96
Gajurel BP et al. 2014 [50]	Mean MRS score at 1 month = 3.58 ± 1.5 mRS 0 = 1/101 mRS 1 = 5/101 mRS 2 = 18 mRS 3 = 34 mRS 4 = 15 mRS 5 = 9 mRS 6 = 19
Jwarchan B et al. 2020 [35]	Association of comorbidities with good outcome mRS 0–1 and poor outcome mRS 2–6 seen
Karn R et al. 2018 [51]	Mean mRS score at 1 month = 2.66 ± 1.803 Mean mRS score at 6 months = 2.71 ± 2.013
Khattar NK et al. 2019 [36]	At discharge, GOS 1 = 12/21 GOS 2–4 = 7/21 GOS 5 = 2/21
Poudel RS et al. 2015 [21]	At 3 months, No symptoms at all (mRS 0) = 4/37 No significant disability (mRS 1) = 9/37 Slight disability (mRS 2) = 7/37 Moderate disability (mRS 3) = 5/37 Moderate severe disability (mRS 4) = 4/37 Death (mRS 6) = 8/37
Shah B et al. 2020 [56]	At 1 month of stroke onset, Total functional dependence = 5/60 Partial functional dependence = 19/60 Independent = 11/60 Total functional dependence = 25/60
Shrestha S et al. 2015 [57]	At 3 months, Independent = 29/56 Dependent = 11/56 Death = 16/56
Shrestha R et al. 2018 [43]	At discharge, mRS 4 = 12/12 At 3 months, mRS 2 = 7/12, mRS 3 = 5/12
Thapa L et al. 2016 [44]	At discharge, mRS 0 = 4/9, mRS 3 = 2/9, mRS 4 = 1/9, mRS 5 = 2/9
Thapa A et al. 2018 [45]	At discharge, mRS < 2 = 558/946 (58.9%) At the end of 3 years, mRS < 2 = 115/130 (88.5%)
Shakya D et al. 2019 [27]	WHODAS mean score = 46.0 ± 23.6 HRQOLISP mean score = 60.6 ± 13.6

## Discussion

Our study is the first systematic literature review to describe the overall picture of stroke patients and stroke care in Nepal and to analyze which aspects of stroke care have been scientifically investigated, what is known from these research results, and where there is an unmet need in research.

Despite a comprehensive search strategy, we identified only 55 studies conducted in Nepal within the last 20 years and analyzed stroke outcomes or aspects of care. The low quantity of studies weighs even more seriously because half of the studies are also of low quality, and there has yet to be a randomized controlled trial (RCT) on stroke care in Nepal. Therefore, the most urgent implication of our work is that more high-quality research is needed.

Most studies were conducted in densely populated areas in central Nepal, with better health infrastructures than in western regions. The studies were all done in tertiary care and teaching facilities and hence may not represent the situation for stroke patients in remote areas or at lower levels of care but rather overestimate the level of stroke care in Nepal. However, as with other diseases, patients from rural communities are referred to tertiary care centers for treatment, and the study population can be said to comprise patients from rural parts of the country. Hence, we need dedicated studies to get an accurate picture of the rural parts of the country. It is even more alarming that even in this setting, no stroke units are described, and the rate of thrombolysis is below 2.5%, so we must assume a thrombolysis rate of less than 1% for Nepal. As IVT, EVT, and stroke unit care are the mainstay of acute therapy in ischemic stroke [65], our systematic review emphasizes the need for dedicated and organized stroke care to improve the country’s overall picture of stroke care.

The mean age of stroke presentation varied from 68.6 years in men to 72.9 years in women [66]. The pooled results in our study showed a mean age of 62.4 years, which is younger than the global average; 63.1 in low-middle income country (LMIC) vs. 68.6 in high-income countries (HIC), which might be attributed to limited stroke care quality and accessibility [67]. Our study showed more men than women suffering from stroke. A systematic review done in 19 countries by Appelros et al. showed stroke incidence to be 30% higher in men than women and 41% more prevalent in men than women [66]. Further research is necessary to understand if this gender gap is caused by a reduced incidence of stroke in women or restricted access to care.

Our review highlighted a high prevalence of preventable risk factors in stroke patients, aligning with other studies’ findings [1, 68–70]. Policymakers should focus on preventing noncommunicable diseases through effective primordial, primary, and secondary prevention strategies [71] and adapt WHO strategies (e.g., Tobacco Control Convention) to meet the needs of Nepal.

The unavailability of stroke units is all the more detrimental because the long-term outcome of stroke can be significantly improved by preventing complications and recurrent stroke, which usually happens in a stroke unit. Pneumonia and UTI were common post-stroke infections (18.8 and 7% of patients) associated with more extended hospital stays. This data is similar to other studies showing UTI and pneumonia as the most common complications [72, 73]. As only four studies reported complications, our pooled data may not accurately portray the country’s real scenario, and more focused studies on post-stroke complications are required. Stroke recurrence is common, especially in large artery atherosclerosis and cardioembolic stroke. While we found a high rate of carotid plaque, only 3% of reported patients had atrial fibrillation, which might be due to insufficient detection methods. Studies on the prevention of complications, secondary prevention, and long-term functional outcome are scarce in Nepal [74].

The economic burden of stroke for an LMIC like Nepal cannot be overstated. The use of thrombolysis treatment ($1390) is expensive, especially under consideration of the annual per capita gross domestic product (GDP) of the country ($1208.22) [75, 76]. In addition, the loss of active earnings by stroke patients’ family members, costs of rehabilitation, and nursing care might even exceed the costs of acute treatment [77]. Hence, cost-effectiveness studies will be crucial to evaluating the direct and indirect costs of stroke care. Further, LMICs like Nepal need to focus more on preventing stroke and imparting knowledge to stroke patients’ families, which can assist significantly in minimizing overall stroke care costs.

### Strengths and limitations

As the first study of its type, this is a milestone in stroke care in Nepal, especially in the absence of a population-based or hospital-based national stroke registry. However, our review has some limitations. We only included the articles in English and could not retrieve some full-text articles. We have also likely not identified all relevant articles published in non-indexed journals. Also, most of the studies included in our review were observational and were of low level of evidence as per Oxford grading. We, therefore, highlight the need to allocate more resources for research and access to publication in international journals for scientists from LMICs.

## Conclusion

Without a national stroke registry, our systematic literature review will be highly relevant to Nepal’s medical community and policymakers. We observed the demographics of stroke patients to be similar to those from other regions, but the provision of stroke care needs to catch up to international standards. Based on the available literature, we highly recommend conducting more high-quality research in Nepal, especially in rural settings outside Kathmandu. Our systematic review emphasizes the absence of structured stroke care in Nepal and the urgent need to improve access to quality stroke care. Hence, with the collaboration of the medical fraternity, local bodies, and government, we must establish stroke care units, educate community members and caregivers, and adapt WHO-tested disease prevention models.

### Electronic supplementary material

Below is the link to the electronic supplementary material.

Supplementary Material 1

Supplementary Material 2

Supplementary Material 3

## Data Availability

All data generated or analyzed during this study are included in this published article and its supplementary information files.
