# Supplementary material for: Stroke epidemiology and outcomes of stroke patients in Nepal: a systematic review and meta-analysis
Source: BMC Neurol. 2023 Sep 25;23:337. doi: 10.1186/s12883-023-03382-5 (PMC10519080; doi:10.1186/s12883-023-03382-5)
Supplement: Supplementary file 1 — Supplementary Material 1 [file 12883_2023_3382_MOESM1_ESM.docx]

## Additional File 1: Search strategy

| Pubmed | (Stroke* OR  TIA* OR  stroke care* OR  stroke outcome* OR  (knowledge* AND stroke*) OR  (risk factor* AND stroke*) OR  (cost* AND stroke*) AND Nepal*) | http://www.ncbi.nlm.nih.gov/pubmed/ |
| --- | --- | --- |
| Ovid MEDLINER | ((stroke* OR TIA*) AND (treatment* OR therapeutics* OR thrombolysis* OR thrombolytic therapy* OR fibrinolytic agents* OR mechanical thrombolysis* OR Emergency Medical Services* OR Emergency Medical Technicians* OR prehospital care* OR Hospitals* OR Rehabilitation* OR Neurological Rehabilitation* OR Rehabilitation Centers* OR Rehabilitation* OR "Physical and Rehabilitation Medicine”* OR Stroke Rehabilitation*) AND Nepal) | https://ovidsp.ovid.com |
| ISI Web of Science | (Stroke* OR  TIA* OR  stroke care* OR  stroke outcome* OR  (knowledge* AND stroke*) OR  (risk factor* AND stroke*) OR  (cost* AND stroke*) AND Nepal*) | https://webofknowledge.com |
| Cochrane Library | (Stroke* OR  TIA* OR  stroke care* OR  stroke outcome* OR  (knowledge* AND stroke*) OR  (risk factor* AND stroke*) OR  (cost* AND stroke*) AND Nepal*) | https://www.cochranelibrary.com |
| Clinical trials.gov | (Stroke* OR  TIA* OR  stroke care* OR  stroke outcome* OR  (knowledge* AND stroke*) OR  (risk factor* AND stroke*) OR  (cost* AND stroke*) AND Nepal*) | https://clinicaltrials.gov |
| Google | Stroke* AND Nepal* | www.google.com |
| Google scholar | Stroke* AND Nepal* | https://scholar.google.com |
