# Supplementary material for: Stroke epidemiology and outcomes of stroke patients in Nepal: a systematic review and meta-analysis
Source: BMC Neurol. 2023 Sep 25;23:337. doi: 10.1186/s12883-023-03382-5 (PMC10519080; doi:10.1186/s12883-023-03382-5)
Supplement: Supplementary file 2 — Supplementary Material 2 [file 12883_2023_3382_MOESM2_ESM.docx]

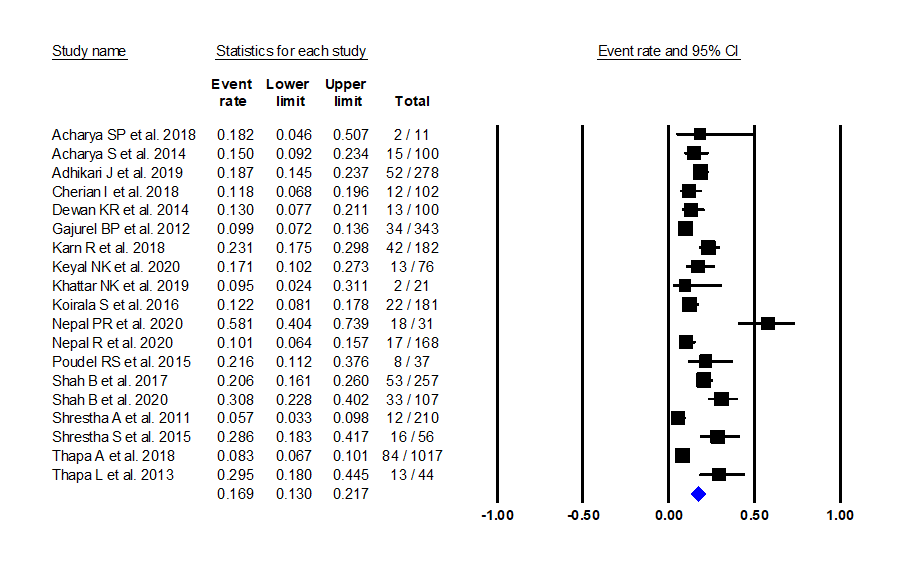


**Figure 1: Mortality rate with all studies included**

**Sensitivity analysis:**


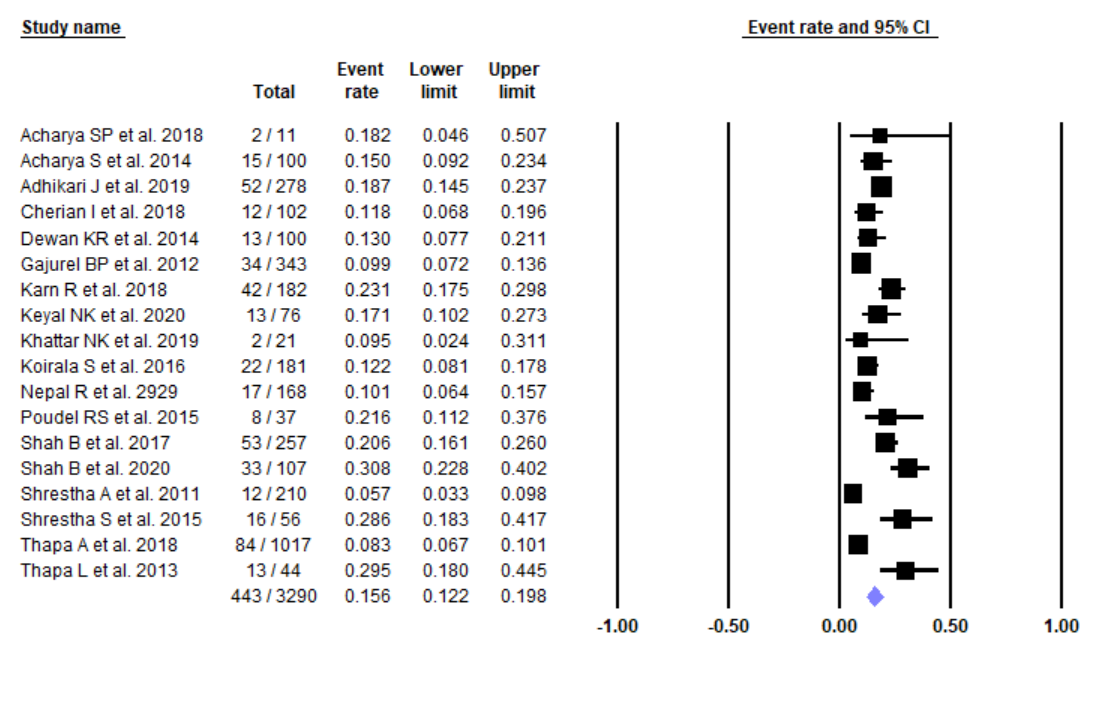


**Figure 2: Mortality rate after removing one study**

Mortality rate after excluding study by Nepal PR et al. 2020 did not show much change in mortality rate. Mortality rate after excluding is 15.6% vs 16.9% when including all the studies.
