# Supplementary material for: Stroke epidemiology and outcomes of stroke patients in Nepal: a systematic review and meta-analysis
Source: BMC Neurol. 2023 Sep 25;23:337. doi: 10.1186/s12883-023-03382-5 (PMC10519080; doi:10.1186/s12883-023-03382-5)
Supplement: Supplementary file 3 — Supplementary Material 3 [file 12883_2023_3382_MOESM3_ESM.docx]

Appendix 3 Education, ethnicity, and job of study participants

| **STUDY** | **Education** | **Profession/Jobs** | **Ethnicity/Location** | **Religion** | **Income** |
| --- | --- | --- | --- | --- | --- |
| Acharya S. et al 2016 | - | - | - | - | - |
| Acharya SP. et al 2018 | - | - | - | - | - |
| Acharya S. et al 2014 | - | - | - | - | - |
| Adhikari J. et al 2019 | - | - | - | - | - |
| Aryal M. et al 2010 | - | - | - | - | - |
| Bhatt VR et al 2008 | - | - | Brahamins:29/61 Chhetri:14/61 Newar:11/61 Others:7/61 | Hinduism: 53/61 Buddhist: 8/61 | - |
| Cherian I. et al 2018 | - | - | - | - | - |
| Chhetri PK. et al. 2012 | - | - | - | - | - |
| Deo RK. et al. 2008 | - | - | - | - | - |
| Devkota KC. et al. 2006 | - | - | - | - | - |
| Dewan KR. et al. 2014 | - | - | - | - | - |
| Dhungana K et al. 2018 | - | - | - | - | - |
| Dhungana K et al. 2019 | - | - | - | - | - |
| Gajurel BP et al. 2012 | - | - | - | - | - |
| Gajurel BP et al. 2014 | - | - | - | - | - |
| Gautam B et al. 2018 | - | - | - | - | - |
| Ghimire RK et al. 2005 | - | - | - | - | - |
| Jha R et al. 2018 | - | - | - | - | - |
| Jwarchan B et al. 2020 | - | - | - | - | - |
| Karn R et al. 2018 | - | - | Himalayan region 8/182 (4.4%) Hilly region 150/182 (82.4%) Terai region 24/182 (13.2%) | - | - |
| Karn R et al. 2015 | - | - | - | - | - |
| Keyal NK et al. 2020 | - | Unemployed 64/191(33.5%) Farmer 52/191(27.2%) Housewife 22/191(11.5%) Student 21/191(10.9%) Labour 6/191(3.1%) Businessman 7/191(3.6%) Technical worker 15/191(7.8%) Army 4/191(2.0%) | - | Hindu 168/191(87.9%) Kirat 15/191(7.8%) Buddhist 5/191(2.6%) Muslim 2/191(1.0%) Christian 1/191(0.5%) | - |
| Khattar NK et al. 2019 | - | - | - | - | - |
| Koirala SR et al. 2016 | - | - | - | - | - |
| Kumari S et al. 2018 | - | - | - | - | - |
| Lamichane BS et al. 2020 | - | - | - | - | - |
| Luitel R et al. 2020 | - | - | - | - | - |
| Maskey A. et al 2011 | - | - | - | - | - |
| Naik M et al. 2006 | - | - | - | - | - |
| Nepal G et al. 2019 | - | - | - | - | - |
| Nepal PR et al. 2020 | - | - | - | - | - |
| Nepal R et al. 2020 | - | - | - | - | - |
| Pahari SK et al. 2013 | - | - | - | - | - |
| Poudel RS et al. 2015 | - | - | - | - | - |
| Rajouria AD et al. 2012 | - | - | - | - | - |
| Roka YB. et al 2011 | - | - | - | - | - |
| Shah SK et al. 2016 | - | - | - | - | - |
| Shah B et al. 2017 | - | - | - | - | - |
| Shah B et al. 2020 | - | - | - | - |  |
| Shakya D et al. 2019 | Illiterate 75/155 (48.4%) <10 years of formal education 56/155 (36.1%) >10 years of formal education 24/155 (15.5%) | Earning 67 (43.2%) Not earning 88 (56.8%) | - | - | <NRs 50,000 122/155 (78.7%) NRs 50,000-1,00,000 27/155 (17.4%) >NRs 1,00,000 6/155 (3.9%) |
| Shrestha A et al. 2011 | - | - | - | - | - |
| Shrestha GS et al. 2012 | - | - | - | - | - |
| Shrestha S et al. 2015 | - | - | - | - | - |
| Shrestha A et al. 2018 | - | - | Brahmin: 6/51(11.8%)  Chhetri: 5/51(9.8%)  Magar: 4/51(7.8%)  Muslim: 3/51(5.9%)  Newar: 5/51(9.8%)  Tharu: 23/51(45.1%)  Others: 5/51(9.8%) | - | - |
| Shrestha R et al. 2018 | - | - | - | - | - |
| Shrestha R et al. 2020 | - | - | - | - | - |
| Shrestha E et al. 2020 | - | - | - | - | - |
| Thakur MK et al. 2017 | - | - | - | - | - |
| Thapa GB et al. 2013 | - | - | - | - | - |
| Thapa L et al. 2014 | - | - | - | - | - |
| Thapa L et al. 2016 | - | - | - | - | - |
| Thapa A et al. 2018 | - | - | - | - | - |
| Thapa L et al. 2013 | - | - | - | - | - |
| Tuladhar AS et al. 2012 | - | - | - | - | - |
| Yadav AK et al. 2020 | - | - | - | - | - |
